# Analysis of DNA methylation profiles during sheep skeletal muscle development using whole-genome bisulfite sequencing

**DOI:** 10.1186/s12864-020-6751-5

**Published:** 2020-04-29

**Authors:** Yixuan Fan, Yaxu Liang, Kaiping Deng, Zhen Zhang, Guomin Zhang, Yanli Zhang, Feng Wang

**Affiliations:** Jiangsu Livestock Embryo Engineering Laboratory, College of Animal Science and Technology, Nanjing Agricultural University, Nanjing, 210095 China

**Keywords:** DNA methylation, WGBS, Skeletal muscle, Development, Sheep

## Abstract

**Background:**

DNA methylation is an epigenetic regulatory form that plays an important role in regulating the gene expression and the tissues development.. However, DNA methylation regulators involved in sheep muscle development remain unclear. To explore the functional importance of genome-scale DNA methylation during sheep muscle growth, this study systematically investigated the genome-wide DNA methylation profiles at key stages of Hu sheep developmental (fetus and adult) using deep whole-genome bisulfite sequencing (WGBS).

**Results:**

Our study found that the expression levels of DNA methyltransferase (DNMT)-related genes were lower in fetal muscle than in the muscle of adults. The methylation levels in the CG context were higher than those in the CHG and CHH contexts, and methylation levels were highest in introns, followed by exons and downstream regions. Subsequently, we identified 48,491, 17, and 135 differentially methylated regions (DMRs) in the CG, CHG, and CHH sequence contexts and 11,522 differentially methylated genes (DMGs). The results of bisulfite sequencing PCR (BSP) correlated well with the WGBS-Seq data. Moreover, Gene Ontology (GO) and Kyoto Encyclopedia of Genes and Genomes (KEGG) functional annotation analysis revealed that some DMGs were involved in regulating skeletal muscle development and fatty acid metabolism. By combining the WGBS-Seq and previous RNA-Seq data, a total of 159 overlap genes were obtained between differentially expressed genes (DEGs) and DMGs (FPKM > 10 and fold change > 4). Finally, we found that 9 DMGs were likely to be involved in muscle growth and metabolism of Hu sheep.

**Conclusions:**

We systemically studied the global DNA methylation patterns of fetal and adult muscle development in Hu sheep, which provided new insights into a better understanding of the epigenetic regulation of sheep muscle development.

## Background

Mutton is a popular meat globally, owing to its low cholesterol, low fat, and high protein content. However, the slow growth rate, low slaughter rate, and low meat yield of sheep in many countries, including China, constitute an important bottleneck that must be addressed to improve the efficiency of large-scale lamb meat production.

The skeletal muscle development is closely related to meat yield and quality in animals reared for meat. The development and growth of muscle involve the proliferation, fusion, and differentiation of myoblast cells into muscle fibers [1]. These processes are affected not only by genotype, but also a set of complicated epigenetic regulatory mechanisms, including DNA methylation. At present, although the mechanism involved in muscle development have been studied at the signaling pathway, transcriptional, and translational levels [2, 3], less is known of the associated epigenetic regulatory mechanisms.

DNA methylation is an epigenetic regulatory mechanism that mediates numerous biological processes such as growth, development, and genomic imprinting [4]. Whole Genome DNA methylation changes in the skeletal muscle have been analyzed based on differentpig breeds, with the results highlighting the differentially methylated regions in the promoter are highly correlated with known obesity-related genes and novel genes, eg. *FTO*, *ATP1B1*, *COL8A2* and so on [5]. Genome-wide DNA methylation profiling in skeletal muscle tissues of aging pigs showed that DNA methylation play a key role in improving proteolysis that is related to muscle function [6]. A comparative analysis of whole genome DNA methylation regulation of gene expressionat the level of transcription in muscles of Japanese Black and Chinese Red Steppes cattle identified several genes associated with DMRs that is related to muscle development [7]. These studies indicate DNA methylation play an important roles in muscle development.

However, little is known about the expression patterns and potential value of DNA methylation in skeletal muscle development of Hu sheep, a Chinese endemic species bred for its meat and skin. The number of sheep muscle fibers increases rapidly at 75–120 d of gestation, following which myofibers grow to fuse and hypertrophy after birth [8]. It is necessary to understand the dynamics of DNA methylation profiles in sheep muscle during these processes. Whole-genome bisulfite sequencing (WGBS) is the most comprehensive DNA methylation sequencing methods available, achieving single-base resolution through bisulfite conversion. WGBS have excellent specificity and non-sensitivity, and can obtain almost complete information of methylcytosine [9]. In our study, we systematically analyzed the DNA methylation profiles in sheep muscle at two key developmental stages (110-day fetus and two-year-old adult) using WGBS technology, thereby expanding the sheep muscle methylome catalog.

## Results

### DNMTs expression levels

**Fig. 1:**
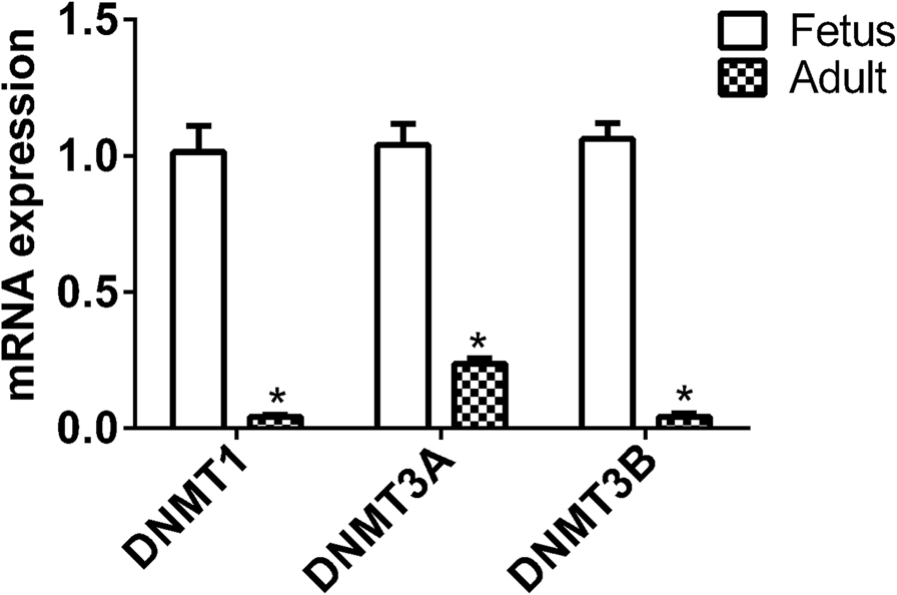
The mRNA expression levels of DNA methyltransferases (DNMTs) determined by qRT-PCR. The relative expression of DNMTs in ovaries was detected by qRT-PCR. The experiment was performed using three biological repeats and three technical repeats. The relative expression levels were normalized to that of *GAPDH*. The results are expressed as means±SEM relative to the fetal samples and the ordinate represents log_10_-transformed values. **P < 0.01

The expression levels of DNMTs (*DNMT1*, *DNMT3A*, and *DNMT3B*) in LD muscle of fetal and adult sheep were first analyzed by Quantitative reverse transcription-PCR (qRT-PCR). The expression levels of *DNMT1*, *DNMT3A*, and *DNMT3B* in the LD muscle of adult sheep were significantly lower than those in fetal LD muscle (Fig. 1) (*P* < 0.05).

### Genome-wide DNA methylation profiling

Global DNA methylation analysis of the LD muscle was performed by WGBS with 30× genome coverage and > 99% conversion efficiency. A total of 78.17 and 75.90 Giga raw bases were generated on average for fetal and adult muscle, respectively. After filtering out low-quality data, approximately 230 million clean reads were generated for each group, with the Q30 of clean, full-length reads ranging from 90.86 to 93.01%. The mapped reads were used for subsequent analysis as the rates ranged from 69.46 to 72.21%. Details of the quality of sequencing data are shown in Table 1.

**Table 1:** Sequencing data by whole genome bisulfite sequencing (WGBS) for sheep Fetus and Adult stages

Groups	Sample	Clean Base (Gb)	Clean Reads	GC(%)	Q30	Mapped (%)	Bisulfite Conversion Rate (%)	Total_mC (%)
**Fetus**	**Fetus1**	78.78	262,822,945	21.54	90.86	69.46	99.72	3.45
	**Fetus2**	79.14	264,029,817	21.93	91.97	71.40	99.73	3.54
	**Fetus3**	76.60	255,561,488	21.85	91.49	70.24	99.74	3.53
**Audlt**	**Audlt1**	70.65	235,680,633	22.09	92.88	71.76	99.73	3.64
	**Audlt**2	72.69	242,505,165	21.84	92.65	72.15	99.72	3.54
	**Audlt3**	84.37	281,442,576	21.93	93.01	72.21	99.70	3.59

**Fig. 2:**
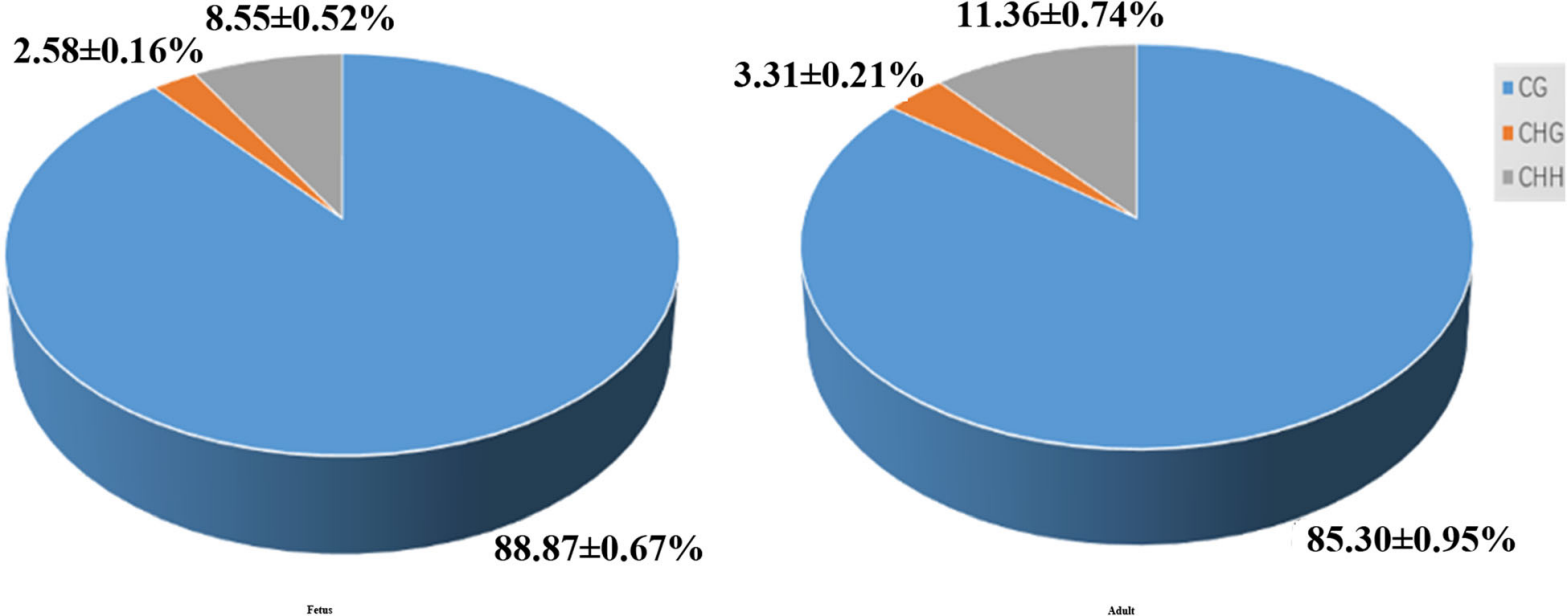
The average ratio of DNA methylation types in fetal and adult genomes of Hu sheep. The blue, orange, and gray colors represent methylated (m) CG, mCHG, and mCHH, respectively

All methylated genomic C sites were approximately 3.5% in each group (Table 1). The CG, CHH, and CHG (where H is A, C, or T) methylation levels were different. We found genome-wide methylated cytosine (mC) levels of 88.87 ± 0.67% for CG, 2.58 ± 0.16% for CHG, and 8.55 ± 0.52% for CHH in fetal samples, and 85.33 ± 0.95% for CG, 3.31 ± 0.21% for CHG, and 11.36 ± 0.74% for CHH in adult samples, and proportions of these contexts were similar in each group (Fig. 2).

**Fig. 3:**
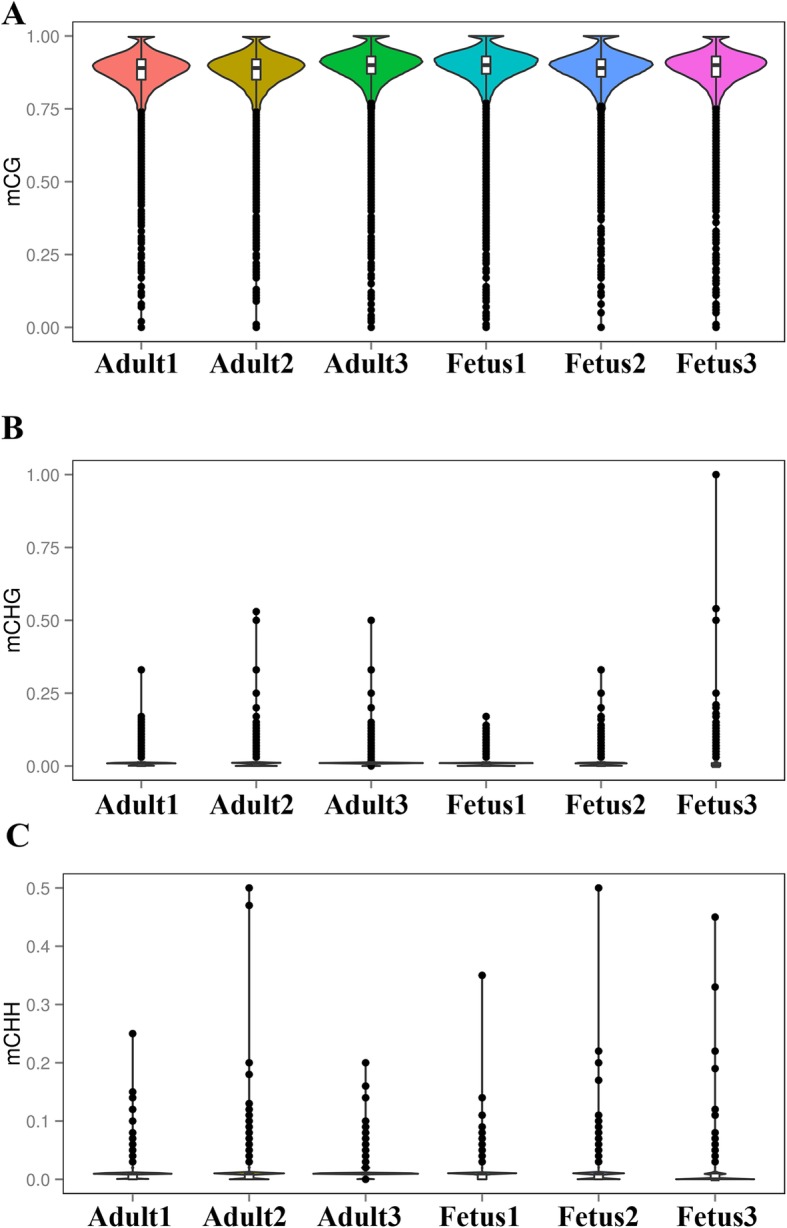
Violin plot for the overall distribution of methylation levels for different methylation types. (**a**) CG, (**b**) CHG, and (**c**) CHH. Fetus (fetus 1, fetus 2, fetus 3), adult (adult 1, adult 2, adult 3). H = A, C or T. The abscissa represents the different samples, the ordinate represents the level of methylation of the samples; the width of each violin represents the density of the point at that methylation level, while the boxplot shows the methylation levels in each violin

A violin graph was drawn with points representing different levels of methylation. The CG methylation levels were high with wide sections in the violin graph (Fig. 3a), but CHG and CHH methylation levels were low with narrow sections in the violin graph (Fig. 3b and c). And then chromosome methylation maps for fetus and adult samples were plotted. The results showed that most chromosomal cytosine hypermethylation was found in the CG context and that the chromosomal mC sites were different between the fetal and adult stages (Additional file 9).

**Fig. 4:**
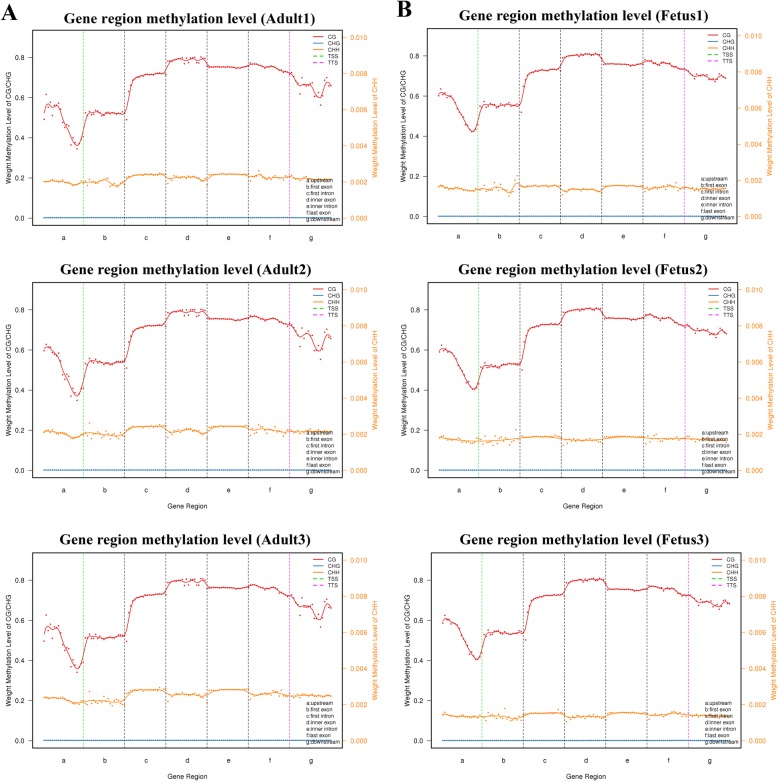
DNA methylation levels across genomic elements in fetal and adult sheep. (**a**) Adult, (**b**) fetus. The abscissa represents different genomic elements, with a, b, c, d, e, f, and g denoting upstream, first exon, first intron, inner exon, inner intron, last exon, and downstream, respectively. The left ordinate represents the methylation levels of CG/CHG contexts, and the right ordinate represents the methylation levels of the CHH context. The dotted, green, vertical line represents the transcription start site (TSS), and the red, orange, and blue solid lines represent CG, CHH, and CHG, respectively

To further compare the genome-wide distribution and the methylation levels of various functional genomic elements between the two developmental stages, we analyzed the methylation status of six different regions, including the promoters, 5′UTRs (untranslated regions), exons, introns, 3′UTRs and distal intergenic. No significant differences were observed among the different genetic elements for the three mC contexts. Overall, the methylation levels in the CG context were higher than those in the CHG and CHH contexts, where the CHH context was hypomethylated and stable in all the functional elements and the CHG context was almost entirely unmethylated. The DNA methylation levels in the CG context were highest in introns, followed by exons (except the first exon) and downstream regions, with sites near the transcription start site (TSS) showing the lowest level. The methylation levels gradually decreased from the promoters to the TSSs and increased from the TSSs to the introns. More detailed information is listed in Fig. 4 and Additional file 3.

### Characterization of DMRs

**Fig. 5:**
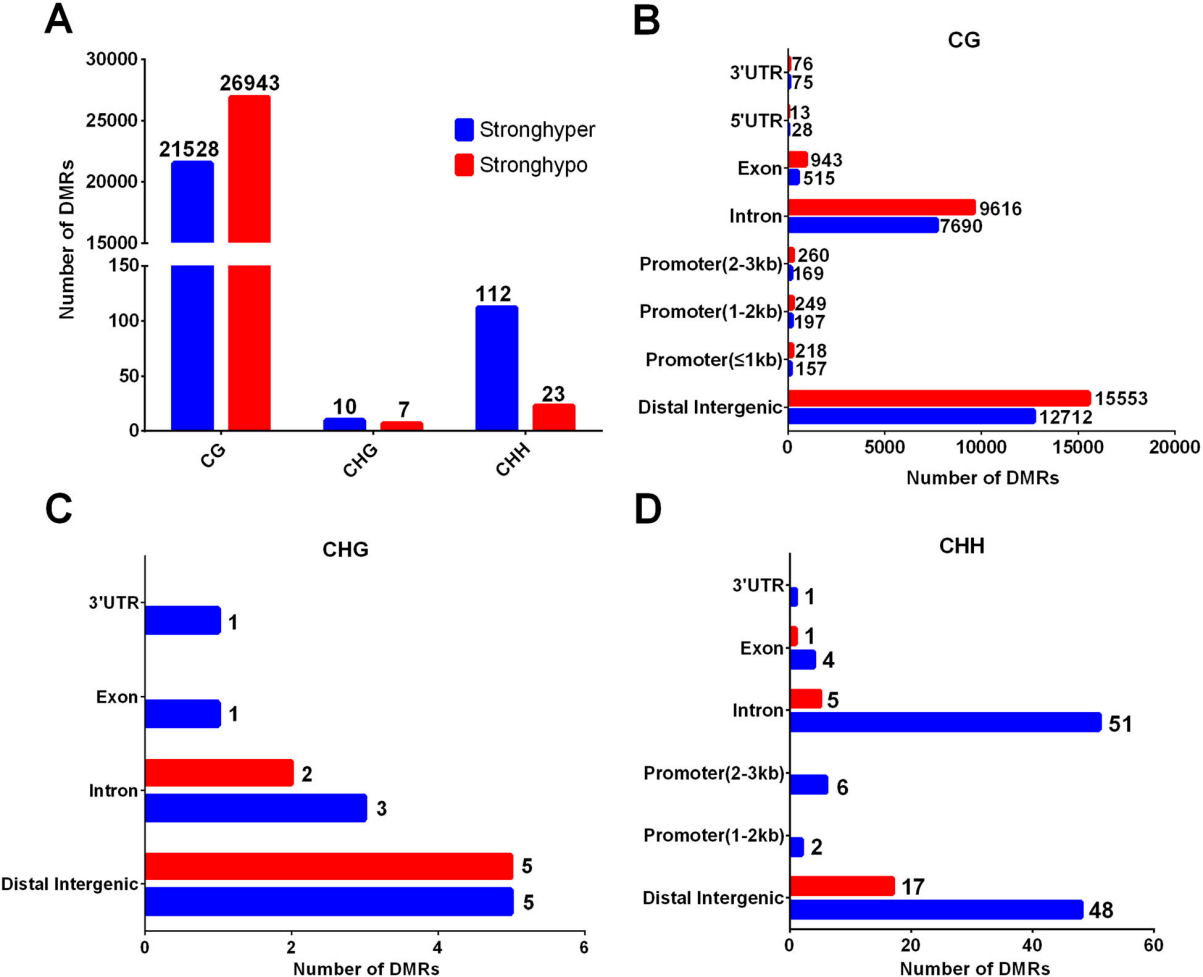
Identification of differentially methylated regions (DMRs) among the fetal and adult sheep muscle samples. (**a**) The number of differentially methylated regions between different methylation types. Histograms show the distribution numbers of DMRs in different genomic elements in the CG (**b**), CHG (**c**), and CHH (**d**) contexts

We had identified 48,491 differentially methylated CG regions, 17 differentially methylated CHG regions, and 135 differentially methylated CHH regions. Among the DMRs, 21,640 (CG:21528 + CHG:10 + CHH:112) were hypermethylated and 26,937 (CG:26943 + CHG:7 + CHH:23) hypomethylated. The DMRs were mostly located at distal intergenic regions, followed by introns, exons, and regulatory regions such as promoters, 5′UTRs, and 3′UTR. In the CG context, only 41,151, and 1250 DMRs were in 5′UTRs, 3′UTRs, and promoters, respectively. More detailed information is listed in Fig. 5.

**Fig. 6:**
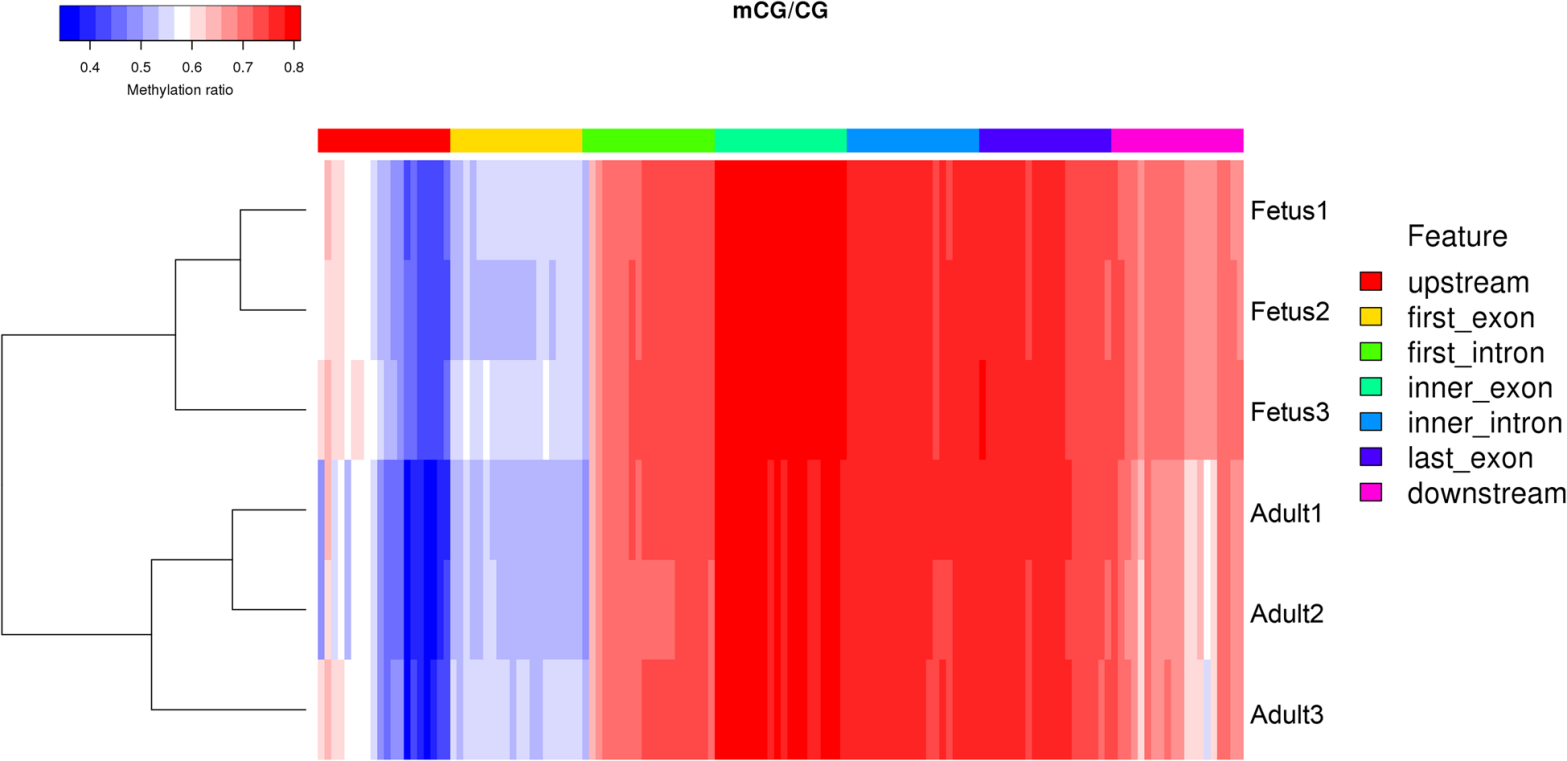
Heat map cluster analysis of differentially methylated regions (DMRs) in different gene functional regions. In the heat map, highly methylated loci are displayed in red and sparsely methylated loci in blue. In addition, the red, yellow, green, turquoise, blue, purple, and pink colors indicate upstream, first exon, first intron, inner exon, inner intron, last exon, and downstream, respectively, and are shown above the heatmap

To detect relatedness between samples individuals, pearson’s correlation coefficient (r^2^) was used as the evaluation index. The results showed the correlation between replicates in each group was high, thus indicating that further data analysis is reasonable (Additional file 10). In addition, as shown in the heat maps in Fig. 6, we analyzed genome-wide methylation in sheep at the fetal and adult stages using hierarchical clustering. The results showed a clear separation between the two developmental stages. More detailed DMR results are listed in Additional file 4.

### Validation of WGBS data by bisulfite sequencing

**Fig. 7:**
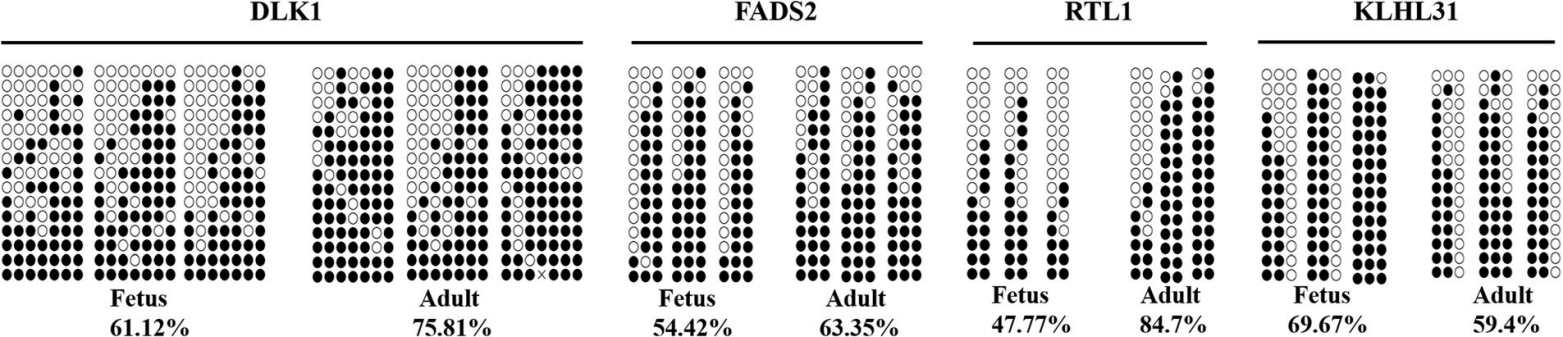
Validation of the whole-genome bisulfite sequencing (WGBS) data by bisulfite sequencing PCR. *P < 0.05, **P < 0.01

To verify the reliability of the WGBS-Seq data, four regions were randomly selected for bisulfite sequencing PCR (BSP). Although the differences in methylation levels among the DMRs (Table 2 and Fig. 7) validated by BSP were lower than those obtained by WGBS, trends were consistent, and the differences might be due to differences in methylation levels of different animals in each stage. On the whole, the BSP results agreed well with the WGBS data, indicating that the WGBS data were reliable and suitable for further study.

**Table 2:** DMR methylation levels of DLK1, KLHL31, FADS2 and RTL1 during muscle development of Hu sheep

Gene	Chr	Start	End	Meth_direction	Meth diff	P-value
DLK1	18	64,325,751	64,325,850	strongHyper	0.31	2.10E-05
FADS2	21	39,753,170	39,753,412	strongHyper	0.418	7.09E-05
RTL1	18	64,485,526	64,485,739	strongHyper	0.334	2.50E-07
KLHL31	20	6,964,690	6,964,822	strongHypo	−0.334	4.73E-14

Note: Chr: chromosome. Meth diff: the difference in methylation levels between Fetus and Adult

### GO and KEGG enrichment analysis of DMGs

**Fig. 8:**
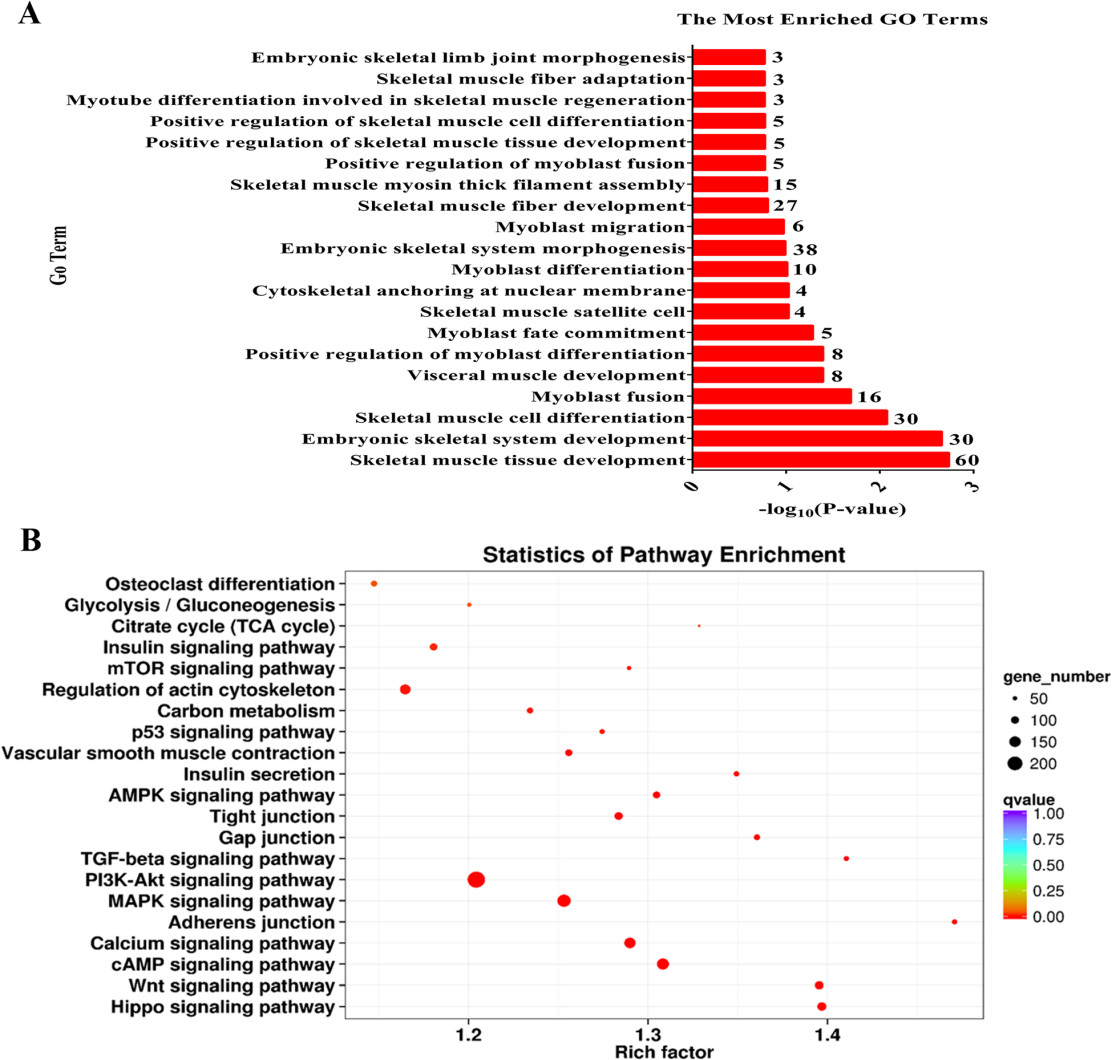
Enrichment analysis of CG-type differentially methylated genes. (**a**) Counts of DMGs enriched for the top 20 muscle development-related Gene Ontology (GO) terms. The abscissa represents the number of DMGs, and the ordinate shows the GO pathway terms. (**b**) The scatterplot of 21 muscle development-related Kyoto Encyclopedia of Genes and Genomes (KEGG) pathways. The ordinate represents the enriched pathways, and the abscissa represents the Rich factor of the corresponding pathways; the size of the spots represents the number of genes related to DMRs enriched in each pathway, while the color of the spot represents the corrected *P*-value for each pathway. The Rich factors indicate the ratio of the number of DMGs mapped to a certain pathway to the total number of genes mapped to this pathway. Greater Rich factor means greater enrichment. DMRs: differentially methylated regions; DMGs: differentially methylated genes

To explore the change in the methylation status of genes under muscle development, the GO and KEGG databases were used to annotate 11,522 DMGs detected in the DMRs. Because most of the DMGs were of the CG context (more than 95%), we focused on CG methylation for the DMG functional enrichment analysis. Based on the GO database, the terms that play a key role in muscle growth and are significantly enriched (corrected *P* < 0.05), including embryonic skeletal system development, skeletal muscle cell differentiation, and skeletal muscle tissue development. The 20 most significantly differentially enriched muscle development-related GO terms for DMGs between fetal and adult LD samples are shown in Fig. 8a. According to the KEGG pathway analysis, DMGs were significantly enriched in the Hippo, cAMP, PI3K-Akt, calcium, and MAPK signaling pathways. The 21 muscle development-related KEGG terms with a corrected *P*-value < 0.05 for the DMGs are listed in Fig. 8b. The results suggest that these DMGs, which are influenced by DNA methylation, can affect muscle development. More detailed results of the COG (cluster of orthologous groups of proteins), GO, and KEGG analyses of CG, CHG, and CHH methylation are shown in Additional file 11-13 and Additional file 5.

### Screening of potential functional differential methylation genes involved in muscle development

**Fig. 9:**
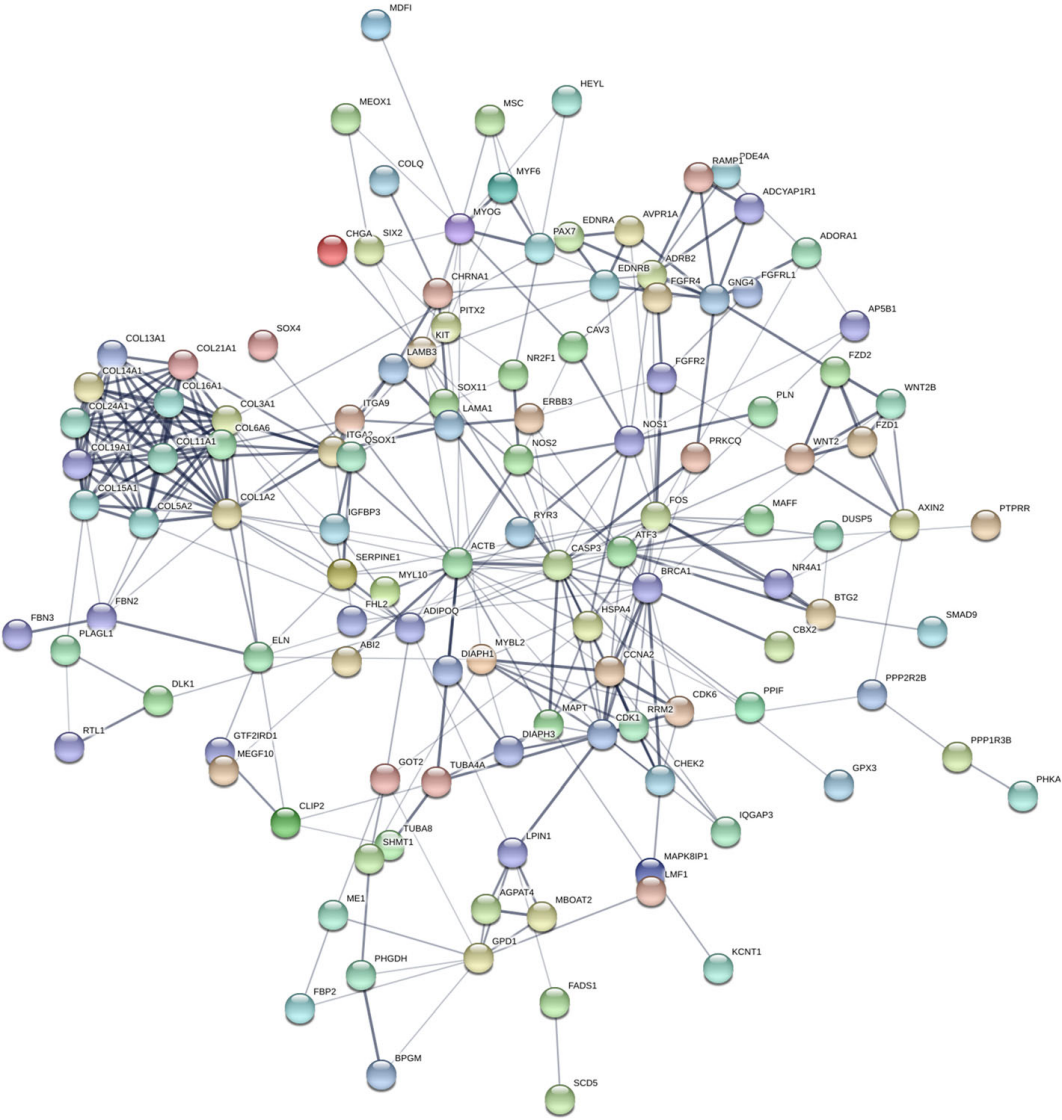
Construction of the network of differentially methylated genes (DMGs) related to muscle development. Analysis of the interaction between DMGs related to muscle development using STRING software according to the interplay index (confidence > 0.7). The interplay index between genes was represented by edge width and transparency. Dark and wide edges indicated high confidence

To identify key genes involved in the regulation of skeletal muscle development, we set three limiting factors to perform an association analysis. First, we screened 1914 candidate genes known to be associated with muscle development through GO and KEGG functional enrichment analysis of significant DMGs (Additional file 6-7). Second, using our previous RNA-seq data between fetus and adult, we further screened 525 overlap genes between DMGs and differentially expressed genes (DEGs). Third, we identified 159 genes with fragments per kilobase of exon model per million reads mapped (FPKM) values > 10 and a fold change > 4, and their interaction network was generated using STRING software. In total, we identified 118 candidate genes that interacted with each other. As shown in Fig. 9, *ADIPOQ*, *CCNA2*, *ITGA2*, *MYOG*, *MAPT*, *DIAPH1*, *NR4A1*, *DLK1*, and *COL1A2* were identified as hub genes in the interaction network related to the muscle development pathway. More detailed results on the abovementioned genes are listed in Additional file 8.

### The regulatory effect of differential gene methylation on the development of sheep muscle

**Fig. 10:**
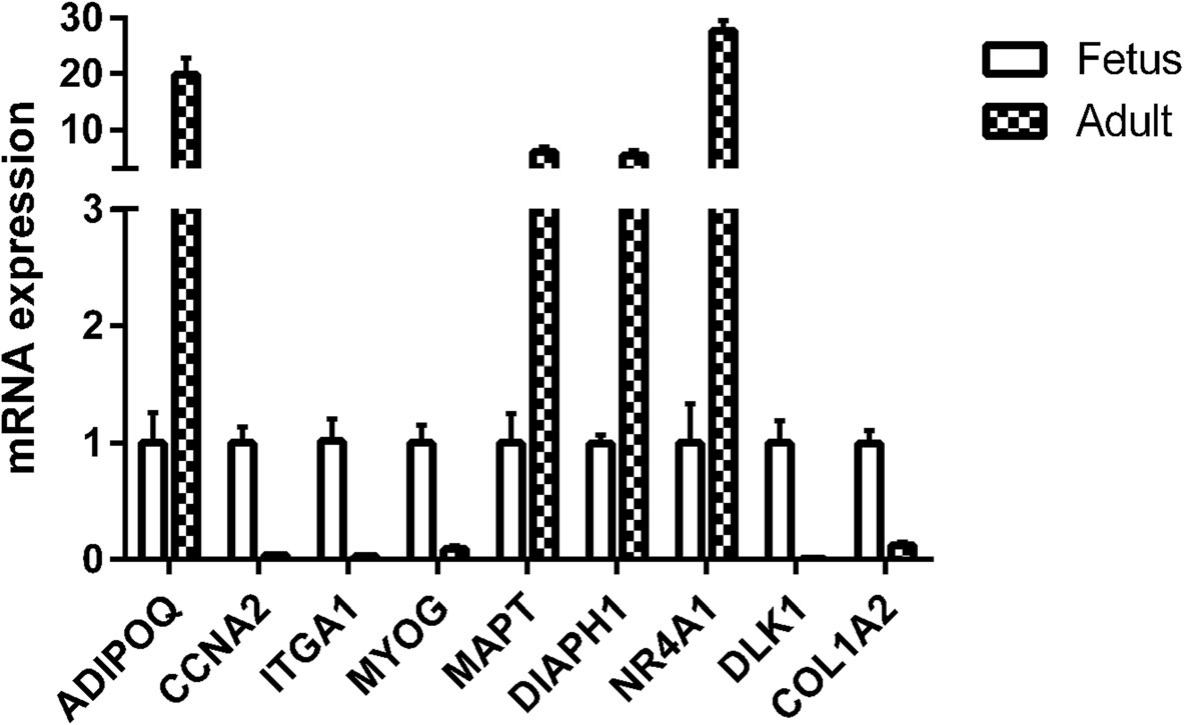
qRT -PCR was performed to detect the relative mRNA expressions of candidate genes in longissimus dorsi (LD) muscle. *GAPDH* was used as internal control. Values are expressed as means ± SEM of three replicates. *P < 0.05, **P < 0.01

To investigate the effect of DNA methylation on gene expression levels, we compared the trend between gene expression and methylation levels using the FPKM value for the RNA-seq data and the difference in methylation levels between fetal and adult WGBS-seq data samples. The results showed that the DMRs of *ADIPOQ* and *ITGA1* genes were in distal intergenic and intron respectively, trends of DNA methylation levels of those genes were consistent with those of expression. Furthermore, qRT-PCR results showed that the expression level of the *ADIPOQ* gene was upregulated with development, while that of *ITGA1* was downregulated. In addition, *DIAPH1* and *NR4A1* genes have DMRs in intron and distal intergenic besides DMRs in promoter regions, the DNA methylation levels of the *DIAPH1* and *NR4A1* genes were downregulated at the adult stage, which was the opposite of that observed for their expression levels. Furthermore, the qPCR results showed that the expression levels of the *DIAPH1* and *NR4A1* genes were significantly upregulated at the adult stage. *DLK1* genes have more DMRs in exon and distal intergenic besides 1 DMR in promoter regions, and *CCNA2* have 1 DMR in intron regions. The DNA methylation levels of the *DLK1* and *CCNA2* genes were upregulated at the adult stage, which was the opposite of that observed for their expression levels. Furthermore, the qPCR results showed that the expression levels of *DLK1* and *CCNA2* were downregulated at the adult stage. As *MAPT*, *MYOG* and *COL1A2* genes may have contained more DMRs in gene body (exon, intron and distal intergenic) and promoter, the DNA methylation levels were inconsistent. And the expression of the *MAPT* gene tended to be upregulated, while that of the *MYOG* and *COL1A2* genes tended to be downregulated at the adult stage. The qPCR results were in good agreement with the RNA-seq data. However, the levels of DNA methylation in the promoter regions of *MAPT*, *DIAPH1*, *NR4A1*, and *DLK1* were the opposite of that observed for their expression levels. The results indicated that DNA methylation in promoter regions of *MAPT*, *DIAPH1*, *NR4A1*, and *DLK1* affected their gene expression levels during skeletal muscle development. While the effect of DNA methylation in gene body regions on *ADIPOQ*, *DIAPH1*, *CCNA2*, *ITGA1* and *COL1A2* genes expression was variable. More detailed information is listed in Table 3 and Fig. 10.

**Table 3:** DMGs most likely involved in muscle development

	RNA-seq data			WGBS-seq data			
Gene name	FPKM of Fetus	FPKM of Adult	log2FC	MethChr	MethDiff (FetusV Adult)	*P-*value	Annotation
ADIPOQ	2.7 ± 0.70	59.02 ± 8.37	5.07	1 (198577572–198,577,648)	0.355	1.95E-13	Distal Intergenic
CCNA2	3.96 ± 1.32	0.22 ± 0.04	−3.53	6 (3651607–3,651,817)	0.494	8.16E-19	Intron
ITGA1	6.3 ± 2.32	0.22 ± 0.06	−4.20	16 (26099670–26,099,819)	−0.374	1.02E-08	Intron
MYOG	118.2 ± 11.46	8.76 ± 1.99	−3.15	12 (196054–196,192)	−0.409	2.76E-07	Exon
				12 (220130–220,377)	−0.314	4.32E-09	Distal Intergenic
				12 (229114–229,232)	0.54	7.28E-07	Distal Intergenic
MAPT	1.08 ± 0.27	6.49 ± 1.04	3.23	11 (45304861–45,304,888)	−0.418	6.01E-08	Distal Intergenic
				11 (45312449–45,312,737)	−0.308	1.53E-10	Promoter (2-3 kb)
				11 (45313729–45,313,893)	−0.34	4.93E-09	Promoter (1-2 kb)
				11 (45317381–45,317,584)	0.399	4.47E-20	Intron
				11 (45327134–45,327,228)	−0.519	6.13E-12	Intron
				11 (45328297–45,328,427)	−0.372	1.50E-07	Intron
				11 (45345547–45,345,760)	−0.368	9.78E-08	Intron
				11 (45360455–45,360,557)	0.371	2.30E-07	Intron
				11 (45379302–45,379,326)	−0.374	1.46E-08	Intron
				11 (45380382–45,380,415)	−0.413	8.46E-09	Intron
				11 (45390429–45,390,641)	0.368	4.67E-06	Intron
				11 (45398919–45,399,057)	0.371	2.28E-08	Intron
				11 (45434897–45,434,935)	0.522	4.15E-08	Distal Intergenic
DIAPH1	1.79 ± 0.12	9.73 ± 1.47	3.08	5 (50071804–50,071,997)	−0.382	2.45E-16	Intron
				5 (50074050–50,074,580)	− 0.343	3.12E-15	Intron
				5 (50078005–50,078,016)	−0.515	9.21E-17	Intron
				5 (50093277–50,093,488)	−0.51	4.68E-11	Promoter (1-2 kb)
NR4A1	1.9 ± 0.64	46.14 ± 5.86	5.20	3 (134110748–134,110,763)	−0.462	4.49E-10	Distal Intergenic
				3 (134112701–134,112,875)	−0.4	1.53E-08	Intron
				3 (134118207–134,118,238)	−0.474	3.23E-06	Promoter (1-2 kb)
DLK1	123.79 ± 16.79	1.59 ± 0.18	−5.62	18 (64227388–64,227,489)	0.315	4.53E-06	Distal Intergenic
				18 (64229987–64,230,060)	0.566	1.12E-11	Distal Intergenic
				18 (64285447–64,285,772)	0.315	2.15E-09	Distal Intergenic
				18 (64297875–64,297,887)	0.509	2.27E-07	Distal Intergenic
				18 (64322164–64,322,397)	0.303	7.14E-08	Distal Intergenic
				18 (64325751–64,325,850)	0.31	2.10E-05	Promoter (2-3 kb)
				18 (64332000–64,332,064)	0.361	5.28E-05	Exon
				18 (64374254–64,374,306)	0.372	2.43E-05	Distal Intergenic
				18 (64374640–64,374,824)	0.567	2.60E-33	Distal Intergenic
				18 (64379476–64,379,559)	0.418	1.43E-05	Distal Intergenic
COL1A2	1509.82 ± 168.72	166.04 ± 40.84	−2.53	4 (11479735–11,479,933)	0.316	1.92E-05	Distal Intergenic
				4 (11513253–11,513,266)	−0.37	2.60E-06	Distal Intergenic
				4 (11551771–11,551,783)	0.433	2.40E-08	Distal Intergenic
				4 (11553777–11,553,809)	0.334	7.76E-06	Distal Intergenic
				4 (11712184–11,712,329)	0.309	5.19E-12	Distal Intergenic

Note: chr: chromosome. DMR: different methylated regions. Meth diff: the difference in methylation levels between Fetus and Adult; a positive number means the methylation levels of this region in the Adult group are higher than those in the Fetus group, and a negative number means that the methylation levels of this region in the Adult group are lower than those in the Fetus group

## Discussion

DNA methylation is a epigenetic regulation form with important roles in gene expression and tissue development [10]. Although muscle DNA methylation has been analyzed in cattle [11], pigs [12], humans [13], mice [10], and sheep [14], genome-wide DNA methylation analysis of sheep muscle has only been performed for specific types of metabolism, in different breeds, at one developmental stage. To our knowledge, this is the first systematic comparison of genome-wide DNA methylation profiles of the LD muscle between fetal and adult Hu sheep.. In our previous study, we confirmed through transcriptome analysis that numerous genes and pathways related to growth and development were differentially expressed between the fetal and adult stages in Hu sheep [15]. Therefore, the genome-wide DNA methylation profile of sheep muscle were investigated by WGBS to elucidate the relationship between differential muscle development and DNA methylation.

DNA methylation is mainly caused by the DNA methyltransferase (DNMT) family, including DNMT1, DNMT3A, and DNMT3B. DNMT1 mainly plays a role in maintaining DNA methylation, while DNMT3A and DNMT3B catalytic de novo methylation. In our study, we found that the expression levels of *DNMT1* and *DNMT3A* in the fetus were higher than in the adult, indicating that DNMTs may play an important role in regulating the transcription of genes related to sheep muscle development.

The muscle of genome-wide methylation patterns were similar between fetus and adult in functional genomic regions. However, there were differences among the three mC contexts which might be related to differences in the sequences of the different genetic elements [7]. After filtering the WGBS-seq raw data, we obtained 230 million clean reads per sample. The rate of uniquely mapped reads ranged from 69.46 to 72.21%, higher than the values found in skeletal muscle satellite cells of mice by MeDIP [10]. Approximately 3.5% of cytosine sites were methylated, with the highest proportion of CG methylation. These results were similar to those found in other species [16] and tissue [17]. Among the gene functional regions, TSSs presented the lowest methylation levels, which was consistent with the results found in sheep ovaries by WGBS [17].

In our study, 48,643 DMRs and 11,522 genes related to these DMRs were identified. Most of the DMRs were small fragments (50–1000 nucleotides; > 90% of the DMRs), suggesting that methylation changes in small regions might be involved in the regulating gene expression. The DMRs were only a small proportion in promoter regions, 3′ UTRs, and 5′UTR, and mainly concentrated in distal intergenic regions (> 60%) with s. To further validate the sequencing results, we randomly selected four regions by BSP, and found that the BSP results were consistent with the sequencing data.

We tried to reveal the roles of DMGs by functional annotations. GO analysis of the DMGs showed that some terms that play a key role in muscle growth, such as skeletal muscle tissue development, embryonic skeletal system development, skeletal muscle cell differentiation, and myoblast fusion. Furthermore, KEGG analysis confirmed that several genes differentially methylated between the two muscle developmental stages might be related to muscle growth. Including Hippo, cAMP, PI3K-Akt, calcium, and MAPK signaling pathways. The PI3K-Akt signaling pathway is critical for skeletal muscle protein synthesis and degradation [18]. The calcium signaling pathway is the key pathway exerting allosteric regulation on many proteins, including through ion channel activation or by acting as a secondary messenger, which could directly affect skeletal muscle metabolism [7]. As muscle is a major metabolic tissue, it was not unexpected that metabolic pathways, including carbon metabolism, insulin secretion, and insulin signaling, were enriched in our study. The bioinformatic results showed that the DMGs related to these regulatory processes show significant differences between fetus and adult, indicating that they may be important for myofiber growth and muscle metabolism. However, how DNA methylation affects gene expression and how these genes work together are still poorly understood.

We generated DMG interaction networks to determine whether DMGs play a determinative role in sheep muscle function. Network analysis showed that *ADIPOQ*, *CCNA2*, *ITGA2*, *MYOG*, *MAPT*, *DIAPH1*, *NR4A1*, *DLK1*, and *COL1A2* were the key nodes.

*DIAPH1* participates in the regulation of cell division, transcriptional activity of serum response factor, and cell motility [19]. DIAPH1 also play a role in signal transduction in smooth muscle cells [20], and DIAPH1 blockade could reduce cardiac muscle cell damage after myocardial infarction [21]. *Dlk1* encodes a transmembrane protein [22], and has been shown to inhibit myoblast proliferation and enhance differentiation when overexpressed in cell culture [23]. The level of *DLK1* mRNA was upregulated in LD in callipyge lambs [24]. Methylation of the *DLK1* gene promoter region increased the invasive ability of non-small cell lung cancer cells [25]. Cyclin A2 (CCNA2) is ubiquitously expressed, and plays an important role in controlling progression through mitosis [26]. Cardiomyocyte mitosis and number were increased in CCNA2-treated pigs [27]. Recent studies have shown that the *ITGA1* gene encodes the integrin receptor alpha 1 subunit and negatively regulates cell proliferation [28]. Moreover, *ITGA1* is important for late muscle differentiation and proliferation in the pig [29]. However, little is known about how DNA methylation of *DIAPH1*, *DLK1*, *ANGPTL4*, *CCNA2*, and *ITGA1* regulates mammalian muscle growth. Several studies have shown that dynamic changes in DNA methylation patterns persist during development and cell differentiation [30, 31]. This suggests that these genes might play important roles in muscle proliferation, and differentiation and regulation of these genes through DNA methylation might be one of the mechanisms determining differential muscle development between the fetus and adult in sheep.

Many of the genes differentially methylated between fetal and adult sheep identified in the present study are also involved in insulin secretion and lipid and glucose metabolism. *ADIPOQ* (encoding adiponectin) is an important regulator of lipid metabolism and glucose and is exclusively secreted from adipose tissue [32]. Short-term fasting can cause significant changes in DNA methylation in the *ADIPOQ* gene promoter in adipose tissue [33]. Moreover, *ADIPOQ* methylation levels were increased by saturated fatty acid overfeeding [34]. In our study, only one DMR (chr1: 198577572–198,577,648, strong hypomethylation in adult sheep) was related to *ADIPOQ* and located in the distal intergenic region, indicating that *ADIPOQ* expression may be influenced by this DMR in the same manner that intragenic DNA methylation can downregulate IGF2 gene expression in cattle [35]. Importantly, the mRNA expression of *ADIPOQ* may determine the responses of follicular to gonadotropins, thereby inducing ovum release. Mutations in the *MAPT* gene are associated with amyotrophic lateral sclerosis [36], while skeletal muscle autophagy can be regulated through *MAPT* [37]. NR4A1, a transcription factor, was shown to regulate the expression of genes involved in wasting the mitochondrial energetic budget and activating the AMPK catabolic pathway [38]. Myofiber size and muscle mass decrease in mice knockout Nr4a1 [39]. In contrast, NR4A1 can promote the expression of genes that control glucose uptake and glycolysis in skeletal muscle [40]. Previous studies have shown that DNA methylation in promoter region were negatively correlated with the NR4A1gene expression level [41]. In addition, the *COL1A2* gene encodes the pro-alpha2 chain of type I collagen, a protein found in most connective tissues. Mutations in *COL1A2* are associated with myopathy [42]. Combined, these results and those from our study indicate that DNA methylation can affect muscle development in sheep.

A complex relationship exists between gene DNA methylation and gene expression levels [7]. Although DNA methylation in promoter regions can inhibit gene expression [43], how DNA methylation within the gene body affects gene expression is poorly understood [44]. In our study, hypermethylation of promoter regions inhibited *DLK1* gene expression, while hypomethylation of promoter regions induced the expression of *MAPT*, *DIAPH1*, and *NR4A1*, consistent with previously reported results [45]. Therefore, we concluded that *MAPT*, *DIAPH1*, *NR4A1*, and *DLK1* were the key regulatory genes during skeletal muscle development, and their DNA methylation status may be the key functional regulators of muscle development. Methylation of these genes may partially contribute to the significant variation observed in muscle development and lipid metabolism between fetal and adult sheep. Nonetheless, the epigenetic mechanisms involved in the regulation of these genes and genetic regions involved in muscle development and lipid metabolism require further study.

## Conclusion

In conclusion, we have provided the first systematic description of genome-wide DNA methylation patterns of sheep muscle at the fetal and adult stages. We investigated several novel and important DMRs/DMGs and pathways related to muscle development in sheep. The results provide valuable data for further understanding the genetic and epigenetic mechanisms that control economic traits in sheep, which could be used to mark assisted selection procedures to promote the growth of skeletal muscle of sheep.

## Methods

### Animals and sample collection

The six ram at fetus (110 days old, 1.36 ± 0.14 kg) and adult (2 years old, 77.98 ± 3.19 kg) stages (*n* = 3) were supplied from Taizhou Hailun Sheep Industry Co., Ltd. (Taizhou, China). The sheep were raised under the same conditions, with natural light and free access to food and water. All animals were fasted overnight and were then euthanized by captive bolt stunning and exsanguination. The LD muscle samples were collected from between the 12th and 13th thoracic vertebrae of the right side at the fetus and adult stages, immediately frozen in liquid nitrogen, and stored at − 80 °C until use.

### Library preparation

DNA was isolated from LD muscle samples using a DNA extraction kit (Tiangen, Beijing, China). The DNA concentration and quality were determined by NanoDrop (NanoDrop Technologies, Wilmington, DE, USA) and agarose gel electrophoresis. Three DNA libraries were constructed for each group. Equal amounts of genomic DNA (2 μg per sample) were fragmented to 400–500 bp by ultrasonication, followed by adenylation and end-repair. The selected fragments were treated with bisulfite and then amplified by PCR to generate the sequencing libraries.

### WGBS and identification of DMRs

The library was sequenced using an IlluminaHiSeqTM2500 platform (Biomarker Technologies, Beijing, China). The peak signal was transformed into sequence data by base calling, following which the raw reads were quality-filtered to obtain the clean reads. First, reads were trimmed of the 3′ adapter sequence. Then, reads with > 10% unknown bases (N) and those of low quality (more than 50% of bases with a PHRED score ≤ 5) were removed. We also calculated the Q30 and GC content.

The clean reads were aligned to the sheep reference genome (Oar_v3.1) and the bisulfite mapping of methylation sites was performed using Bismark software. The duplicates were reads that aligned with the same region of the genome, and can estimated the sequencing depth and coverage. The bisulfite conversion rate is the percentage of methylated clean reads to the total number of clean reads in the genome. The binomial distribution test for each C site was used to confirm C-site methylation by screening conditions for coverage ≥4× and false discovery rate (FDR) < 0.05.

To identify the differentially methylated regions (DMRs) between fetal and adult samples, we referenced the model of [16] to estimate the methylation level. All C sites with read coverage > 10× were used for DMR analysis with MOABS [46]. Subsequently, DMRs were defined by the presence of at least three methylation sites in the region, and in which the difference in methylation levels was > 0.2 (> 0.3 for the CG context) and the *P*-value from Fisher’s exact test was < 0.05.

### Functional enrichment analysis

The DMR-related genes (DMGs) were compared against functional databases such as GO and KEGG by BLAST for annotation of gene function. GO enrichment analysis of the DMGs was implemented by the GOseq R packages based on the Wallenius non-central hypergeometric distribution [47]. KOBAS software was used to test the significance of DMR-related gene enrichment in the KEGG pathway analysis [48]. Pathways with a corrected *P-value* < 0.05 were considered to be significantly enriched. The STRING database was used to analyze interaction networks of selected DMGs (http://string-db.org/) [49].

### Quantitative reverse transcription-PCR

The expression levels of DNA methyltransferase-related genes and validate the DMGs by qRT-PCR. Total RNA was isolated from LD muscles using Trizol reagent (Invitrogen, Carlsbad, USA). cDNA was reverse transcribed from total RNA using the PrimeScript RT kit (Takara, Dalian, China). qPCR was performed on a StepOnePlus Real-Time PCR System (Life Technologies, USA) using SYBR Green Master Mix (Roche Applied Science, Mannheim, Germany). The gene primers are listed in Additional file 1. The relative expression of each gene was normalized to that of *GAPDH* using the 2^−ΔΔCt^ method [50].

### Bisulfite sequencing PCR

The bisulfite sequencing PCR was used to validate DNA methylation levels of selected candidate genes. Genomic DNA was modified with sodium bisulfite using the EZ DNA Methylation-Gold™ Kit (ZymoResearch, Los Angeles, USA). Then, bisulfite-converted gDNA was subjected to PCR amplification using Zymo Taq™ DNA polymerase (ZymoResearch). The PCR products were purified using a Gel Extraction Kit (Shenggong, Shanghai, China), ligated, and cloned into the pUC18-T vector (Shenggong). Fifteen clones of each sample were randomly selected for DNA sequencing. The quantification tool for methylation analysis was used to analyze bisulfite sequencing data (QUMA; http://quma.cdb.riken.jp/). Gene sequence-specific primers are listed in Additional file 2.

### Association analysis

We previously screened many genes related to muscle development at two stages of Hu sheep (fetus and adult) using the Illumina platform, [15]. By association analysis of the differentially methylated genes and the differentially expressed genes, a set of differentially methylation DEGs at the intersection of the two was obtained. Negative correlations between DMR methylation level and the corresponding gene expression level were identified by correlation analysist (*r* with a negative value).

### Statistical analysis

Statistical analyses were performed by the independent samples *t*-test with the SPSS 25.0 software package (SPSS Inc., Chicago, IL, USA). Results of the qRT-PCR data were expressed as means ± standard error of the mean (SEM) of three samples with three biological replicates. Differences were regarded as significant at *P* < 0.05.

## Supplementary information

**Additional file 1.** Primers for qRT-PCR.

**Additional file 2.** Primers sequences of DNA methylation-related genes.

**Additional file 3.** DNA methylation levels in gene functional elements in the Adult group and Fetus group.

**Additional file 4.** Information of DMRs in the Fetus group and the Adult group.

**Additional file 5.** Information about the COG, GO and KEGG analyses of DMGs. DMGs: Differential methylation genes.

**Additional file 6.** The list of DMGs enriched for muscle development-related top 20 GO terms.

**Additional file 7.** The list of DMGs enriched for muscle development-related KEGG terms.

**Additional file 8.** Muscle development -related DMGs target genes in muscle between Fetus and Adult Hu sheep.

**Additional file 9.** Plot of genome chromosome 5-methylcytosine map. A, CG type. B, CHG type. C, CHH type. H = A, C or T.

**Additional file 10.** The correlation coefficients analysis of samples. The closer the number is to 1, the stronger the correlation.

**Additional file 11.** GO and KEGG pathway analysis in CG type DMGs. A, GO analysis. B, KEGG analysis.

**Additional file 12.** COG, GO and KEGG pathway analysis in CHG-type DMGs. A, COG analysis. B, GO analysis. C, top GO. D, KEGG analysis.

**Additional file 13.** COG, GO and KEGG pathway analysis in CHH-type DMGs. A, COG analysis. B, GO analysis. C, top GO. D, KEGG analysis. E, top KEGG.

## Data Availability

The full WGBS-data sets have been submitted to NCBI BioProject under Accession: PRJNA622418. The original data of RNA sequencing has been uploaded to GEO database, the number is GSE101669. Additional data can be found in Additional files.

## Abbreviations

BSP: Bisulfite sequencing PCR
COG: Cluster of orthologous groups of proteins
DEGs: Differentially expressed genes
DMGs: Differential methylated regions related genes
DMRs: Differential methylated regions
DNMT: DNA methyltransferase
FPKM: Fragments per kilobase of exon model per million reads mapped
LD: Longissimus dorsi
FDR: False discovery rate
GO: Gene Ontology
GAPDH: Glyceraldehyde-3-phosphate dehydrogenase
KEGG: Kyoto encyclopedia of genes and genomes
mC: Methylated cytosine
qRT-PCR: Quantitative reverse transcriptase PCR
TSS: Transcription Start Site
UTR: Untranslated regions
WGBS: Whole-genome bisulfite sequencing
