# Supplementary material for: Analysis of DNA methylation profiles during sheep skeletal muscle development using whole-genome bisulfite sequencing
Source: BMC Genomics. 2020 Apr 29;21:327. doi: 10.1186/s12864-020-6751-5 (PMC7191724; doi:10.1186/s12864-020-6751-5)
Supplement: Supplementary file 1 — Additional file 1. Primers for qRT-PCR. [file 12864_2020_6751_MOESM1_ESM.docx]

**Additional file 1:** Primers for qRT-PCR

| Primer Names | Sequences (5’-3’) | | | Product Length(bp) | Gene Bank |
| --- | --- | --- | --- | --- | --- |
|  | **Forward** | | **Reverse** |  |  |
| GAPDH | GTCAAGGCAGAGAACGGGAA | | GGTTCACGCCCATCACAAAC | 232 | NM_001190390.1 |
| DNMT1 | CCTGACTCCACCTACGAAGACC | | TCTACTTGCTCCACCACGAACT | 128 | NM_001009473.1 |
| DNMT3A | AAGCAGGGCAAAGACCAGCATT | | AGCGAAGAGGTGGCGGATGA | 189 | XM_012166008.2 |
| DNMT3B | CGCAGATCAAGCTCACGACT | CGGTTGGAGGTACTGCTGTT | | 203 | XM_004014479.3 |
| ADIPOQ | CCTCTGGCTCCGTGCTCCTC | AGGAAGCCTGTGAAGGTGGAGTC | | 124 | NM_001308565.1 |
| CCNA2 | ACCACAGCACGCACAACAGTC | AGTGTCTCTGGTGGGTTGAGGAG | | 87 | XM_027970780.1 |
| ITGA1 | TACAGTTGACCAATCGGCAGCATC | ATCATAGGCTCCTACGGCTCCAAG | | 109 | XM_027980075.1 |
| MYOG | GGACCCTACAGATGCCCACA | GGTTTCATCTGGGAAGGCCG | | 94 | NM_001174109.1 |
| MAPT | AGAAGCAGGCATTGGCGACAC | CTTCGTTCCAGGCTTACCATCCG | | 145 | XM_027974381.1 |
| DIAPH1 | AGTCCCTTCGTGTCTCCCTCAAC | CCTCCAGCAGTCTCCTCCTTCTC | | 127 | XM_015096089.2 |
| NR4A1 | GCACCTTCATGGACGGCTACAC | GGATGTGGAGGAGGCTGAGGAG | | 107 | XM_027967295.1 |
| DLK1 | GCCCATGGAGCTGAATGCTT | CGGGAAAGGTCACACACTGG | | 124 | XM_015102053 |
| COL1A2 | CCGAGGGCAACAGCAGATTCAC | TCAAGGATAGGCAGGCGAGATGG | | 124 | XM_004007726.4 |
