# Supplementary material for: Analysis of DNA methylation profiles during sheep skeletal muscle development using whole-genome bisulfite sequencing
Source: BMC Genomics. 2020 Apr 29;21:327. doi: 10.1186/s12864-020-6751-5 (PMC7191724; doi:10.1186/s12864-020-6751-5)
Supplement: Supplementary file 2 — Additional file 2. Primers sequences of DNA methylation-related genes. [file 12864_2020_6751_MOESM2_ESM.docx]

**Additional file 2:** Primers sequences of DNA methylation-related genes

| Region | Sequences (5’-3’) | Product Length(bp) | Associate Gene | Gene Bank |
| --- | --- | --- | --- | --- |
| 64325751-64325850 | TTYGGTGGATTTGGAATATTTG | 179 | DLK1 | NC-019475.1 |
|  | AACTCTTAATTACATACTTATCAAAACAC |  |  |  |
| 39753170-39753412 | TATTAAAAGAATGGTTTTGTGTAGG | 310 | FADS2 | NC-019478.1 |
|  | AAATAAATTACCATTCCTTTCTCC |  |  |  |
| 64485526-64485739 | TTTTTGATTAAAGTTTTTTTTGTTT | 272 | RTL1 | NC-019475.1 |
|  | TTTATAAAAAAAAAATAAATTAATCTATATAAC |  |  |  |
| 6964690-6964822 | GAGGTTTATGTATATATTTTTTGTAAGTG | 213 | KLHL31 | NC_019477.1 |
|  | CCTACAAAAATTTTCACTAATTTTTC |  |  |  |
