# Supplementary material for: Analysis of DNA methylation profiles during sheep skeletal muscle development using whole-genome bisulfite sequencing
Source: BMC Genomics. 2020 Apr 29;21:327. doi: 10.1186/s12864-020-6751-5 (PMC7191724; doi:10.1186/s12864-020-6751-5)
Supplement: Supplementary file 3 — Additional file 3. DNA methylation levels in gene functional elements in the Adult group and Fetus group. [file 12864_2020_6751_MOESM3_ESM.docx]

**Additional file 3.** DNA methylation levels in gene functional elements in the Adult group and Fetus group.

**%**

| **Group** | **Context** | **upstream** | **first_intron** | **inner_intron** | **last_intron** | **first_exon** | **inner_exon** | **last_exon** | **downstream** |
| --- | --- | --- | --- | --- | --- | --- | --- | --- | --- |
| Fetus | CG | 64.25767 | 86.48933 | 91.72133 | 91.538 | 62.03833 | 93.02033 | 90.065 | 86.91133 |
|  | CHG | 0.476667 | 0.518667 | 0.518667 | 0.511667 | 0.440333 | 0.48 | 0.494667 | 0.482333 |
|  | CHH | 0.464 | 0.523 | 0.516333 | 0.503667 | 0.425667 | 0.442333 | 0.473667 | 0.476 |
| Adult | CG | 63.68267 | 86.049 | 91.745 | 91.59467 | 60.736 | 92.79167 | 89.863 | 87.05467 |
|  | CHG | 0.650333 | 0.767667 | 0.774667 | 0.749333 | 0.591333 | 0.679333 | 0.701 | 0.704667 |
|  | CHH | 0.685 | 0.822333 | 0.828667 | 0.793 | 0.598667 | 0.721667 | 0.738667 | 0.755 |

Note: The results use numbers to represent the average levels of DNA methylation in each gene functional element.
