# Supplementary figures and images for: Analysis of DNA methylation profiles during sheep skeletal muscle development using whole-genome bisulfite sequencing
Source: BMC Genomics. 2020 Apr 29;21:327. doi: 10.1186/s12864-020-6751-5 (PMC7191724; doi:10.1186/s12864-020-6751-5)

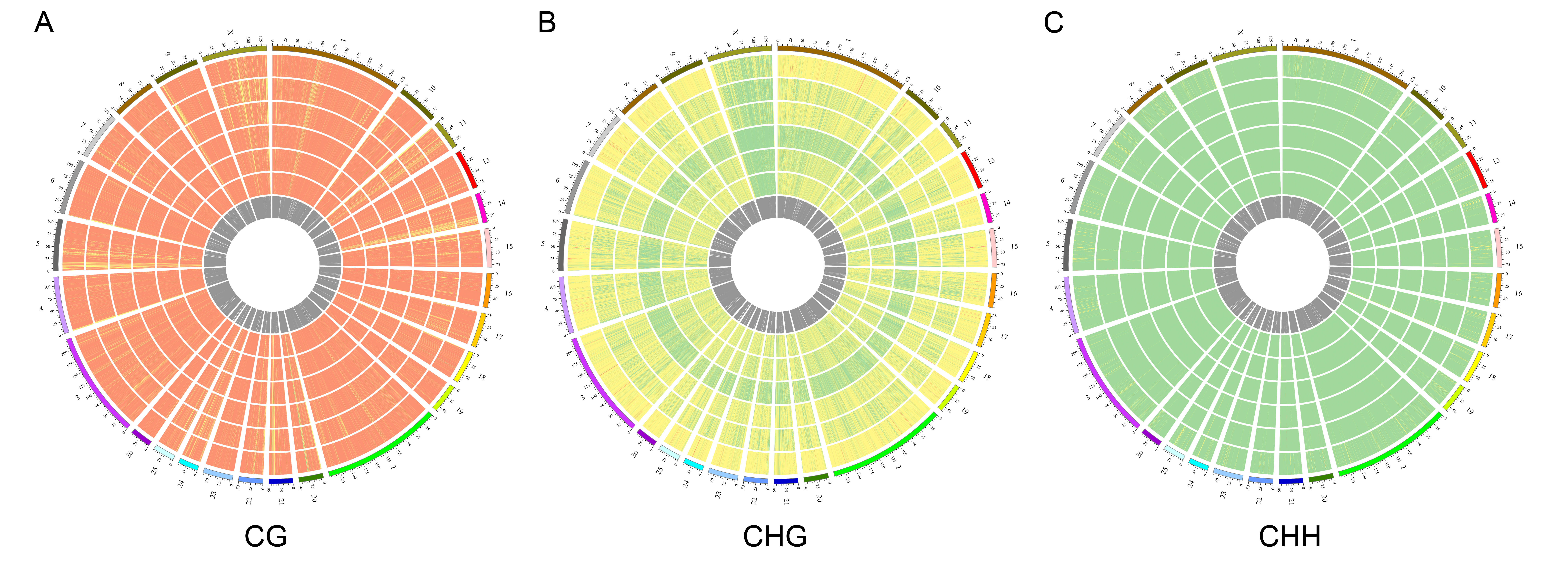

Supplement: Supplementary file 9 — Additional file 9. Plot of genome chromosome 5-methylcytosine map. A, CG type. B, CHG type. C, CHH type. H = A, C or T. [file 12864_2020_6751_MOESM9_ESM.jpg]

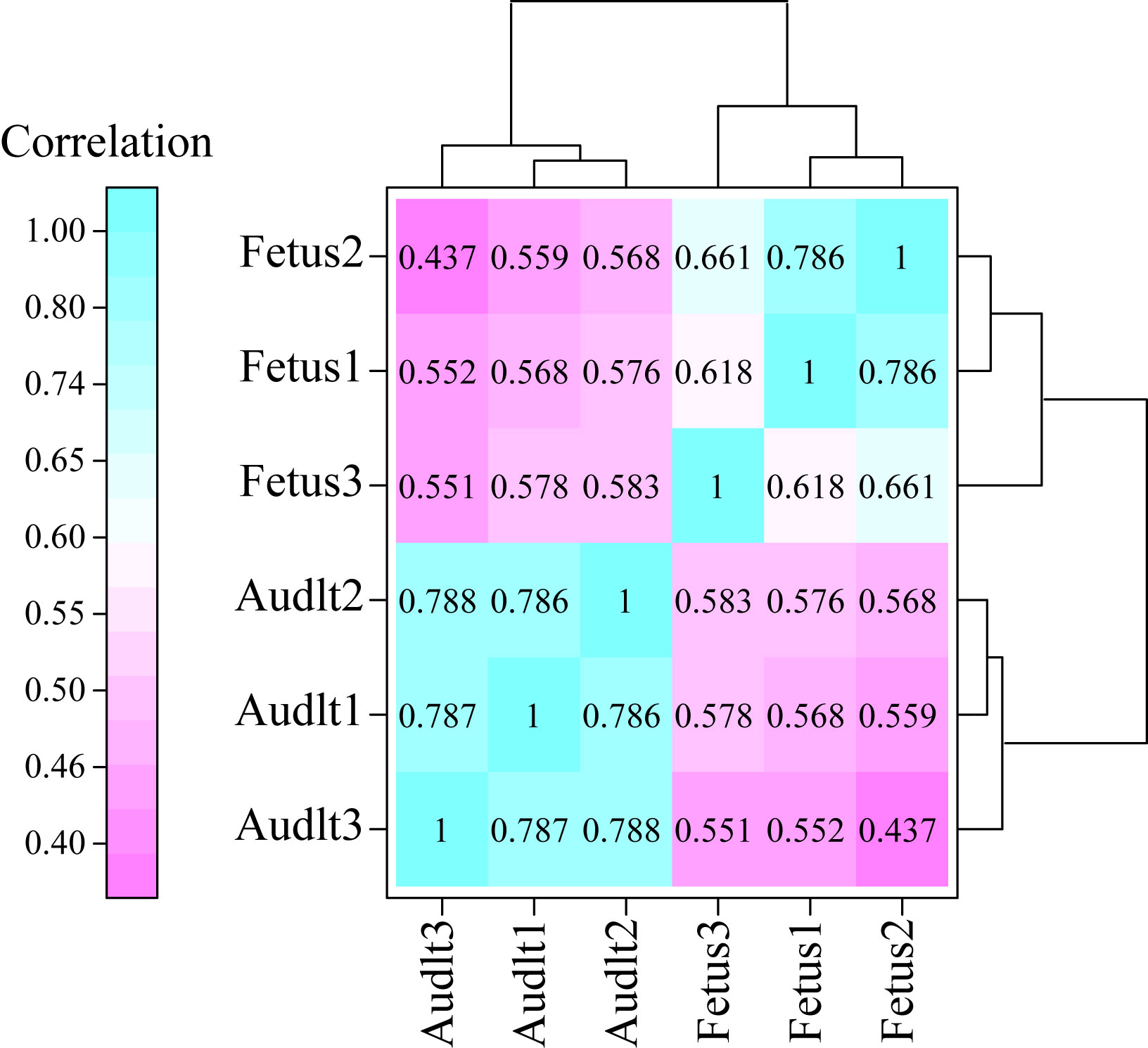

Supplement: Supplementary file 10 — Additional file 10. The correlation coefficients analysis of samples. The closer the number is to 1, the stronger the correlation. [file 12864_2020_6751_MOESM10_ESM.jpg]

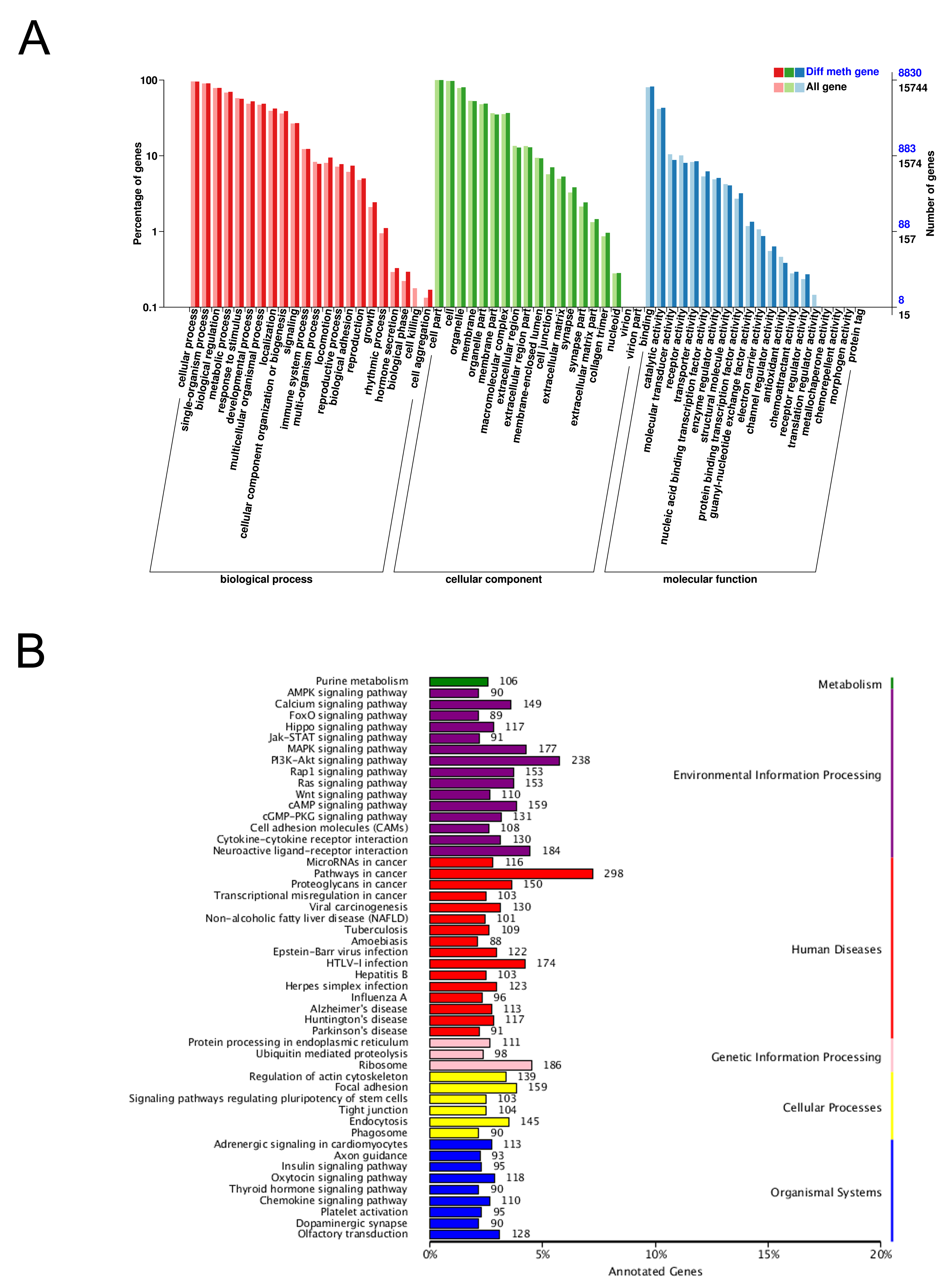

Supplement: Supplementary file 11 — Additional file 11. GO and KEGG pathway analysis in CG type DMGs. A, GO analysis. B, KEGG analysis. [file 12864_2020_6751_MOESM11_ESM.jpg]
